# Measuring the Flex Life of Conductive Yarns in Narrow Fabric

**DOI:** 10.3390/mi14040781

**Published:** 2023-03-30

**Authors:** Paula Veske, Frederick Bossuyt, Filip Thielemans, Jan Vanfleteren

**Affiliations:** Centre for Microsystems Technology (CMST), Interuniversity Microelectronics Centre (IMEC), Ghent University, 9000 Ghent, Belgium

**Keywords:** wearables, e-textiles, electronics, testing, reliability

## Abstract

Due to constant advancements in materials research, conductive textile-based materials have been used increasingly in textile-based wearables. However, due to the rigidity of electronics or the need for their encapsulation, conductive textile materials, such as conductive yarns, tend to break faster around transition areas than other parts of e-textile systems. Thus, the current work aims to find the limits of two conductive yarns woven into a narrow fabric at the electronics encapsulation transition point. The tests consisted of repeated bending and mechanical stress, and were conducted using a testing machine built from off-the-shelf components. The electronics were encapsulated with an injection-moulded potting compound. In addition to identifying the most reliable conductive yarn and soft–rigid transition materials, the results examined the failure process during the bending tests, including continuous electrical measurements.

## 1. Introduction

**Figure 1:**
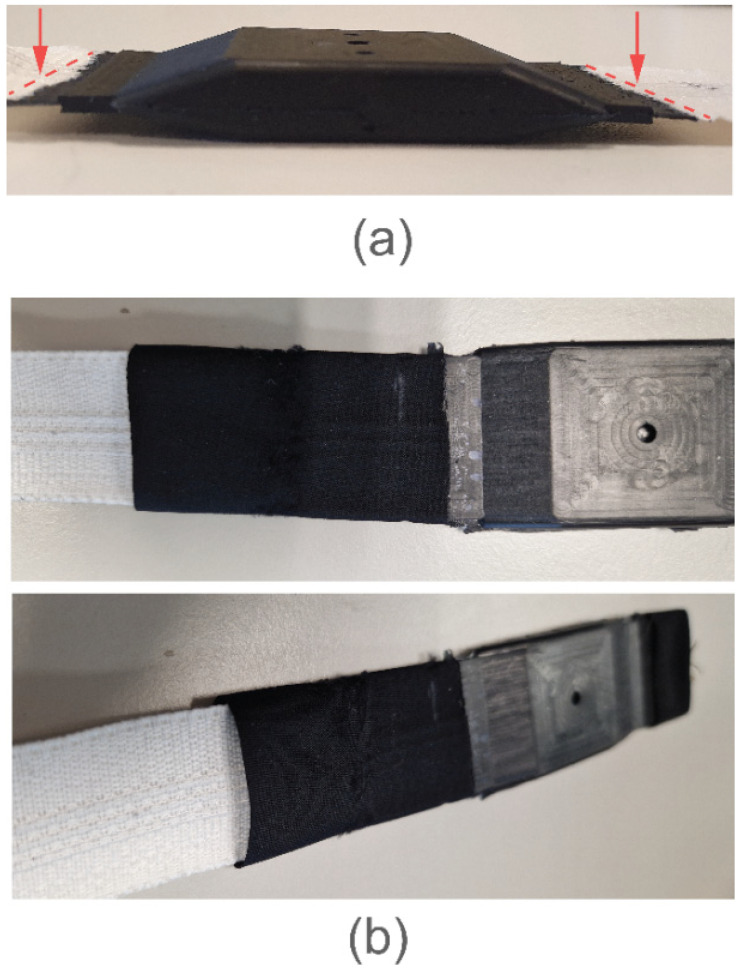
(**a**) Sample with encapsulation; red markings highlight the soft–rigid transition/high-stress areas. (**b**) Sample with additional transition material.

Electronic textiles (e-textiles) have gained increasing traction since the late 20th century [1,2,3,4]. Mann (1996) [3] discussed the advantages of his wearable computer in 1996 and Farringdon et al. (1999) [1] developed a wearable sensor jacket using advanced knitting techniques to form fabric stretch sensors and measure upper limb and body movement. Conductive yarns are an essential part of e-textile applications [5,6]. Nevertheless, integrated electronics often create high-stress areas at the electronics encapsulation–textiles transition points [7,8,9]. For example, de Vries and Cherenack (2014) [7] used conductive yarns (Elektrisola litze wires) woven into polyester fabric to connect LEDs that were mounted to the yarns with isotropic conducting adhesive. The sample underwent cyclic bending, and the authors concluded that the transition area between the LEDs’ connection to the yarns (soft–rigid transition area, see more in Figure 1a) was the clear location of failure.

Thus, measuring conductive yarns’ or textiles’ abilities to withstand repeated bending is essential. Contrary to domestic washing tests, bending tests help to identify the exact moment(s) at which conductive yarns break, thus helping to compare their durability more accurately and under uniform conditions. In this study, the final application for this type of e-textile system is technical and functional work wear worn in extreme conditions. The conditions include high-temperature stress and elevated humidity levels. Thus, durability, reliability and electrical stability are highly important and essential.

This work presents two different conductive yarns woven into polyester-based narrow fabric and a bending testing method together with a self-assembled tester machine constructed from off-the-shelf components. The conductive yarns (commercially available, from Bekaert) were woven into narrow fabric in six rows with a pitch of 1.27 mm to create a bus system for I^2^C communication. A non-functional PCB (printed circuit board) was encapsulated with potting material on the narrow fabric, creating rigid–soft (encapsulation–textile) transition areas and accumulated stress for the conductive yarns (Figure 1a). Several material stacks around the rigid–soft transition area were also designed and developed to reach the highest durability for e-textile systems.

The main goals of this work were to identify:

1. How and when the two conductive yarns would break during repeated bending stress;
2. How the encapsulation material and its transition would influence the yarns;
3. If and how additional transition materials could help the durability of the system.

The results included an analysis of the continuous electrical measurements through the conductive yarns during the tests. A comparison of the durability results was performed mainly between yarn compositions, filament types, encapsulation and soft–rigid transition areas. Moreover, the effects of the encapsulation material’s design and processing, together with additional transition material stacks, are discussed. 

## 2. Materials and Methods

The materials were divided into two categories: testing samples and testing machine. The methods explain the testing process in more detail.

### 2.1. Samples

The testing samples were based on a white narrow fabric into which conductive yarns were woven. The narrow fabric’s total width was 30 mm. The yarns integrated into the narrow fabric were commercially supplied by Bekaert and their listed application areas included data and energy transfer, and use in antennas, sensors or other purposes. The yarns were integrated into the narrow fabric in 6 rows with a pitch of 1.27 mm to create a communication bus for the I^2^C (Inter-Integrated Circuit) communication protocol. Two different yarns were tested during this work, as shown in Table 1. Elongation and tensile strength information were taken from the supplier’s datasheets.

A CO_2_ laser was used to create an opening in the yarn for an electrical connection with electronics. A rigid PCB (printed circuit board) was glued onto the narrow fabric and connected to the conductive yarns by soldering. 

**Figure 2:**
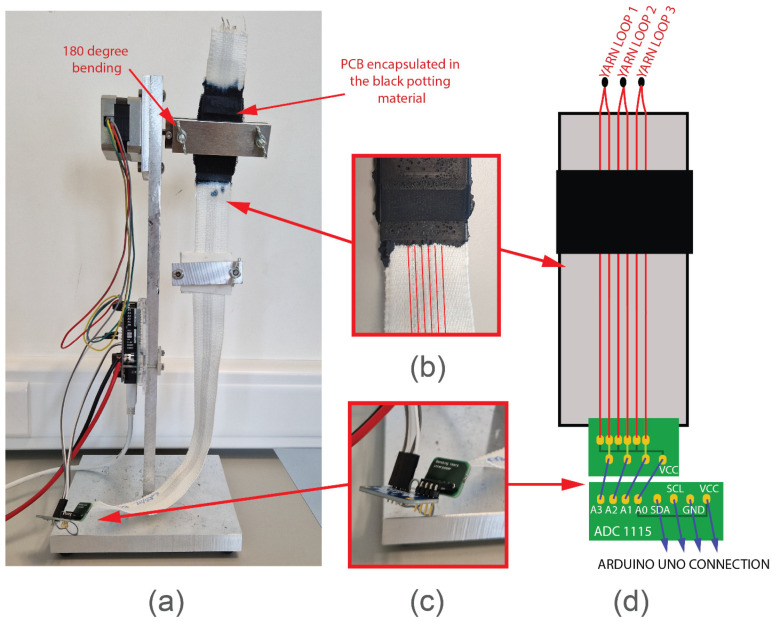
Testing set-up with the motor at the top left side and where the 180-degree rotation also takes place. (**a**) Machine set-up highlighting where the 180-degree bending takes place, where the PCB is placed on the woven ribbon and then encapsulated in the black potting compound, the soft–rigid transition area and the connection between the ADC and sample. (**b**) Close-up photo of the soft–rigid transition area (from the potting compound to the white narrow fabric) also highlighting the conductive yarns woven into the white narrow fabric (in red). (**c**) Close-up photo of the connection between the ADC and the sample. (**d**) Technical drawing of the sample set-up for the bending test: light-grey part represents narrow fabric, red lines represent conductive yarns woven into the ribbon, black part represents the encapsulation/potting and green parts represent the ADC connection to the ribbon.

The integrated electronics were encapsulated with a polyurethane-based flexible potting compound (Peters ELPECAST^®^ VU 4443/92 WR-NV) via the low-pressure injection-moulding process. The potting material was mixed together from two parts. The mixed material was then degassed in a vacuum chamber and injected into the PTFE mould. After injection, the material was cured at 100 degrees for 4 h. The encapsulation was designed to have a smooth transition from thick to thin. The PTFE mould was structured in a way that, at the electronics’ position, the potting material was 8 mm thick and at the transition point from the potting material to the textile, the thickness was 1.5 mm. The different thicknesses were smoothly brought together with a slope—see more in Figure 1a and Figure 2b. However, the difference in the encapsulation material and textile flexibility properties created transition points with higher mechanical stress. The encapsulated sample and highlighted stress areas are shown in Figure 1a.

The integrated conductive yarns in the white narrow fabric and the encapsulation transition area were tested and with additional transition material by wrapping it around the narrow fabric (see Figure 1b). The material was TPU (thermoplastic polyurethane—Bemis 3914, 100 µm) laminated knit fabric (68% microfibre polyamide and 32% elastane). The laminated fabric was wrapped around the narrow fabric and laminated before encapsulating the electronics with potting compound. The fabric did not cover any interconnection points, such as the solder contact points, as they were all encapsulated by the black potting compound. 

A complete overview of the sample set-ups is provided in Table 2. The first sample set included narrow fabric with integrated Yarn 1 (Table 1), together with electronics and their potting encapsulation. There was no additional transition material added. Sample set 2 was the same, but Yarn 2 (Table 1) was integrated into the narrow fabric instead of Yarn 1. Sample sets 3 and 4 corresponded to sample sets 1 and 2, but there was additional transition material (mentioned in the previous paragraph) added to the potting encapsulation edges.

The conductive yarn endings on each side were stripped from the PTFE (polytetrafluoroethylene) cover to form the connection to the read-out system through ADC (analogue-to-digital converter). On one side, the conductive yarn endings were coupled and soldered to create 3 loops (Figure 2d top side). On the other side, the conductive yarn endings were soldered on an interposer PCB with female pins (Figure 2c,d). The female pins led to the ADC board and further on to the Arduino Uno connection. One end of the yarn loop was soldered to the power connection and the other end to the signal connections. See more about the testing method and the read-out system in Section 2.3.

### 2.2. Machine

The tester was developed using off-the-shelf components and a specially milled frame (Figure 2). The fixed part of the machine consisted of:

- A stepper motor Nema 17;
- An Arduino UNO microcontroller together with a Motorshield V2 board;
- An Adafruit ADC1115 (analogue-to-digital converter) together with male 90-degree pins;
- The stepper motor was attached to the top and connected to the Arduino UNO/Motorshield V2 board.

The connections between the Motorshield and Arduino board did not allow the connection of the sample to the microcontroller. Thus, the ADC board was connected in between to read out the data. The male pins on the ADC board allowed the easy connection and disconnection of the sample to the machine.

### 2.3. Testing Method

A sample with a weighted clamp (100 g) was attached from the (black) encapsulated part to the motor and flexed 180 degrees. As the yarns were coupled together, one ending of the loop received power input and the other end was used to read the output. During the test, the samples were flexed 180 degrees for 100,000 cycles or until the voltage of at least two yarn loops (readings) dropped and was not regained for 10 s. The ten-second time period still allowed the yarns to regain some stable connection, but if it was not possible, the yarn/sample was considered failed.

The ADC was used to convert analogue signals to digital signals to read every voltage value measured, not only if the signal was there or not. If the yarn was damaged in between, it would be indicated by the voltage drop. The data read-out included the voltage of the yarns (V), the number of cycles performed and the time (s). There was one bending cycle in one second (60 measurements per minute), and one voltage/resistance measurement was taken during that time. 

The data transmission was read out and saved using PuTTY (an open-source terminal emulator) through the serial port [10]. The data were logged in a CSV (comma-separated values) file that was easily usable for analysis.

However, resistance rise is often a more desired measurement for failure analysis. Resistance can be measured using Ohm’s law by first defining the current (I). 

The original resistance of a yarn (R_Y_) loop was 1.2 Ω. The voltage input was always 5 V and the original voltage output (V_out_) was always 4.95 V. Thus, the voltage loss (V_loss_) at the start was always 50 mV. Based on this, the current was calculated:

(1) $$I=\frac{V_{\mathrm{loss}}}{R_{Y}}=\frac{50\;\mathrm{mV}}{1.2\;\Omega }=41\;\mathrm{mA}$$

When the resistance of the yarns stays within 10% of the load resistance, the current stays stable and it can be assumed to be the same. To calculate the load resistance (R_L_), the following formula can be used:

(2) $$R_{L}=R_{Y}\times \frac{V_{\mathrm{out}}}{V_{\mathrm{loss}}}=1.2\;\Omega \times \frac{4.95\;V}{0.05\;V}=120\;\Omega$$

Thus, if R_Y_ changes to be over 12 Ω, then the current will also change. When yarn resistance changes to be over 12 Ω during the tests (voltage drops), the resistance should be calculated accordingly:

(3) $$R_{y}=\frac{\left(5\;V-V_{\mathrm{out}}\right)\times R_{L}}{V_{\mathrm{out}}}$$

**Figure 3:**
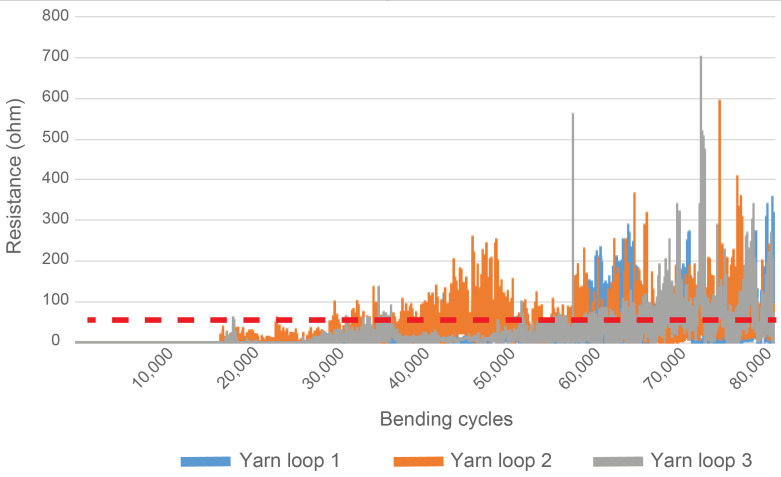
Results of Yarn 1 from sample set 1 (Ni-plated copper yarn) with the red dotted line highlighting the failure points. The figure visualises the whole test of one of the samples to provide an understanding of how the resistance changed over during the test.

Thus, the resistance of the yarn loops can be also plotted during failure analysis (Figure 3). 

## 3. Results

**Figure 4:**
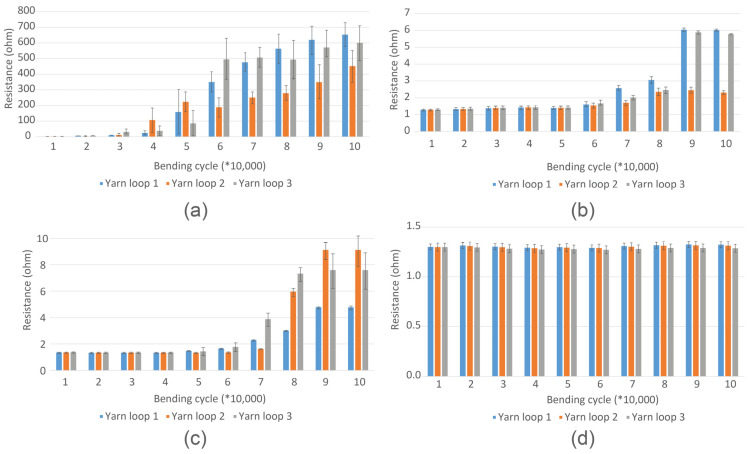
Overview of the results displaying the average resistance of each 10,000 (*10,000) cycle set. (**a**) Sample set 1 with Yarn 1. (**b**) Sample set 2 with Yarn 2. (**c**) Sample set 3 with Yarn 1. (**d**) Sample set 4 with Yarn 2.

As mentioned earlier, samples were flexed 180 degrees for 100,000 cycles or until the voltage dropped (resistance rose) in at least two yarn loops for 10 s over the set limit after bending. Failure points were based on the final needs of the work wear application and its read-out electronics. Voltages below 3.5 V or resistance rises to 50 ohms were considered failure points (shown with red dotted lines in Figure 3 and Figure 4a). 

Firstly, it was observed how earlier resistance rises were reduced back to the original measurements (Figure 3 between 20,000–30,000 cycles). The stable connections were lost due to the filaments breaking during the bending tests. However, it was still possible for the yarn to regain some connection with other filaments, which also resulted in a regain of voltage output. Resistance increases in size and persistence when filaments break. It was observed that yarn with a larger number of filaments (Yarn 2) tended to regain its original conductivity more, was more stable and started to break later. Thus, higher numbers of filaments together with lower sensitivity to plastic deformation under the bending of the base material (steel or copper) support stable connections for longer.

Moreover, the copper-plated steel yarn’s (Yarn 2) durability was considerably better than that of the Ni-plated copper yarn (Yarn 1). The resistance rise was lower and more stable, indicating how steel and a larger number of filaments provide much better durability than nickel-plated copper yarns. Additionally, when one yarn loop broke during the tests, the second failure appeared quite quickly (for Yarn 1).

Figure 4 shows an overview of the results showing the average resistance of the sample sets for each 10,000 cycle set. The greatest deviation in data was observed for sample sets 2 and 3 as only one sample out of four in each case showed failing measurements towards the end of the test. In sample set 1, all the samples failed between 16,000 and 41,000 cycles. In sample set 4, none of the samples failed and they stayed stable until the end of the test.

The resistance rise tended to start gradually and then rise more drastically after some time. For example, in sample set 1 (Figure 4a), the resistance in yarn loop 3 was steady until 50,000 cycles and then it jumped by 450 ohms, indicating that the yarn had broken. Similarly, in sample set 3 (Figure 4c), the resistance in yarn loop 2 was steady until 70,000 cycles and then it rose by 3 ohms. The sudden increase in resistance was less obvious in sample sets 2 and 4 with Yarn 2; however, it was observed in sample set 2 (Figure 4b) at 90,000 cycles.

It was observed that additional transition material helped to increase the bending durability significantly (Figure 4b,d). While sample sets 2 and 3 still contained one sample with failure measurements, overall, the samples remained relatively stable. In sample set 4, no samples showed failure measurements at any time.

## 4. Discussion

In this work, two conductive yarns that were woven into narrow fabric were tested for repeated bending at the soft–rigid electronics encapsulation transition position. The failure points were decided based on the needs of the read-out electronics of the e-textile system. The application of the tested e-textile samples was to finally integrate them into work wear used in extreme conditions, such as high temperature and humidity levels. The e-textile system must, therefore, be highly durable, reliable and electrically stable. 

Thus, this paper focused on three research questions:

1. How and when the two conductive yarns would break during repeated bending stress;
2. How the encapsulation material and transition from it would influence the yarns;
3. If and how additional transition materials could help the durability of the system.

**Figure 5:**
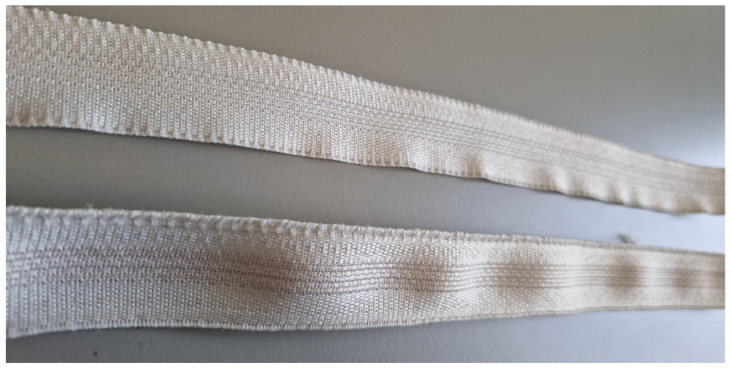
Top: Narrow fabric with 6 rows of Yarn 1. Bottom: Narrow fabric with 6 rows of Yarn 2.

**The conductive yarns’ durability to repeated bending.** Both conductive yarns were woven into polyester-based narrow fabric. In each sample, the ribbon was 30 mm wide and the pitch between the yarns was 1.27 mm. This work did not examine how different narrow fabric widths and/or the pitch of the conductive yarns influenced the final durability. However, while the integration of the yarns in the fabric was exactly the same in either case, the stiffness of the yarns was very different. The drapability of the narrow fabric with Yarn 1 was considerably better than that of the fabric with Yarn 2 (Figure 5). While the filaments of Yarn 2 were steel-based, which also influenced the stiffness of the overall ribbon, this aspect may have impacted the final durability results in at least two ways:

- A stiffer fabric is more difficult to process as it pushes the rigid electronics and encapsulation away from the fabric during processing, thus significantly influencing the final integration;
- Restricting the movement of the fabric during bending, thus creating a larger bending radius and protecting the yarns.

The composition of the yarns also differed. Yarn 2 with steel performed better than the mainly copper-based yarn (Yarn 1). However, based on the supplier’s datasheet, the tensile strength and elongation percentage were lower for Yarn 2. This would suggest that, in the current bending test, these characteristics mattered less. In the current test, the time spent under stress was very short and repeated many times, as opposed to a single long stress test.

**Figure 6:**
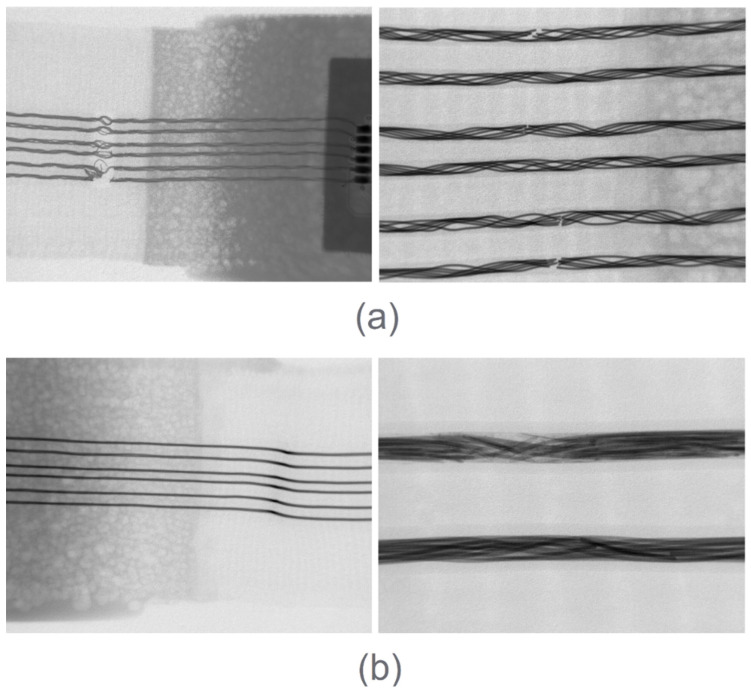
X-ray inspection photos after bending tests. (**a**) Yarn 1 after bending tests. (**b**) Yarn 2 after bending tests.

The best-performing samples were found in sample sets 2 and 4 with Yarn 2. Along with the material’s structure, Yarn 1 had fewer filaments than Yarn 2, which also seemed to have an influence on durability (Figure 6). Moreover, the PTFE coating was 0.04 mm thicker and the filaments were thinner in Yarn 2 than those in Yarn 1 (Table 1). A thinner conductive material has also been seen to be beneficial in previous works, especially around the soft–rigid transition area [11,12,13,14]. X-ray inspection also showed how the filaments of Yarn 1 tended to break completely, while only some Yarn 2 filaments broke entirely, leading to the conclusion that Yarn 2 had higher durability to repeated bending, even at the soft–rigid transition area.

While sample sets 2 (with Yarn 2 without additional transition) and 3 (with Yarn 1 with additional transition) exhibited improved results compared with sample set 1, the standard deviation showed high variability. From both sample sets, one sample out of four failed towards the end of the testing (70,000–90,000 cycles) and created a large gap with the results for the other samples. Thus, this indicates the possibility of failure after 60,000 cycles (see more in Figure 4). 

The more drastic resistance rise patterns were seen in sample sets 1 and 3 with Yarn 1, which could also be explained by the thinner PTFE coating and fewer and thicker filaments. However, as mentioned before, the influence of the steel material is quite clear in Yarn 2’s higher performance. The drastic resistance rise also indicated the complete breakage of the yarn, as also seen in Figure 6a, while in Yarn 2 (Figure 6b), only some filaments seemed to break.

**The effects of the electronics encapsulation material and methods.** The encapsulation material was a polyurethane-based potting compound mixed from two parts. The material’s shore hardness was rated between 55 and 65 (similar to a pencil eraser’s softness). The mixture was also degassed in a vacuum chamber prior to use. However, the pressure produced by the pump was not high enough and the degassing process was either too long (the material curing process already started) or some air bubbles were left in the material. Air bubbles being left in the material made it more porous and may have been an important factor in the final outcome as well. For example, the higher porosity of the potting compound could have caused the transition of the thinner potting compound area to break more easily, thus causing insufficient protection and a rough transition around the soft–rigid materials transition. Moreover, the thicker and thinner encapsulation moulding areas differed by around 6 mm. Although the transition from thicker to thinner areas was designed with a smooth slope (see Figure 1a), the large cap could have also affected the overall durability of the conductive yarns.

The encapsulation material’s higher rigidness compared with the textile was clear by testing sample sets 1 and 3 (without additional transition materials, Figure 4a,b). Yarn 1 started to break more immediately (around 20,000 cycles) and the yarn’s resistance rose drastically, showing the complete breakage of the filaments. Yarn 2 had more durability, but the sudden, more extreme resistance change was still seen, showing how some filaments were breaking. 

Overall, it can be concluded that the smooth and softer transition from the encapsulation to the yarns/textile cannot be underestimated. It is essential to focus on the material characteristics, together with the processing results. The transition points at the soft–rigid transition area and within the encapsulation area need to be designed with high awareness. 

**The effects of the additional transition materials and methods.** Due to the higher rigidness of the encapsulation material, some softer additional material stacks were added to the transition area (Figure 1b). 

It was clear that the durability under constant bending was improved with that addition (Figure 4c,d). The resistance rise was less abrupt or lacking at all (sample set 4). Thus, the added material stack contributed significantly to increasing the reliability and durability of the current e-textile system.

**General overview.** The results indicated that both the conductive yarns’ material and construction affected their reliability and durability. The steel-based yarn with a higher number of filaments, thinner filaments, no core and thicker PTFE coating seemed to have considerably higher durability. Moreover, the encapsulation material characteristics, together with the final outcome after processing, could have also influenced the soft–rigid transition areas due to the porosity of the material. The overall thickness design of the moulded encapsulation may have also contributed to the final durability.

The present results are significant in at least two major aspects. These outcomes confirm the high need for a soft and smooth transition from rigid to soft materials. The additional transition material stack added between the moulded encapsulation and textile had a major influence on the final durability and reliability of the samples (Figure 4c,d). Additionally, the results showed how a high encapsulation level could still be achieved locally for e-textiles. The current e-textile system stack is intended to be used in extreme working conditions where the reliability of the electronics is essential. Thus, the encapsulation of the functional elements tends to be higher than usual, leading to more extreme soft–rigid transition points on the textile. However, this work demonstrates that the transition areas can be improved immensely by introducing soft material stacks in these crucial areas.

## 5. Conclusions

This work introduced a bending tester aimed to determine conductive yarns’ breaking points, focusing on the rigid–soft transition points between the yarns and the electronics’ encapsulation. The tool presents an opportunity to test for proof-of-concept without ordering expensive machines and tests. The testing method was used to bend two different conductive yarns woven into a narrow fabric. The results show how the two yarns degraded differently and at what point they started to degrade. 

Overall, this study demonstrated that the local encapsulation of electronics on textiles can be achieved with a high level of quality. In order to achieve high durability levels, smooth and soft transitions between rigid and soft materials are crucial. The tests also demonstrated that narrow fabric stacks with softer transition areas (from rigid encapsulation) are key to improving durability. Additionally, the composition, number of filaments, thickness and encapsulation greatly affect the reliability of conductive yarns. 

Future work will include alternative testing features, such as a slower or faster bending cycle or a different weight clamp. Additionally, different potting compounds with softer shore hardness levels and different moulding designs may be examined. Moreover, twisting (similar to a crumple test) could be investigated to further understand the mechanical stress influences in e-textile systems. 

## Figures and Tables

**Table 1:** Overview of conductive yarns used in tests.

Characteristic	Yarn 1	Yarn 2
Yarn construction	6 twisted filaments with PE core	14 twisted filaments
Filament diameter	0.075 mm	0.063 mm
Conductive material	Ni-plated Cu (Nickel-plated copper)	Cu-plated steel yarn
Coating material and thickness	PTFE (Polytetrafluoroethylene); 0.12 mm	PTFE; 0.16 mm
Outer diameter	0.60 mm	0.68 mm
Tensile strength (N)	100	>27
Elongation (%)	3.02	0.5–1.5
Average resistance	0.8 ohm/m	0.99 ohm/m

**Table 2:** Sample sets’ set-up.

Sample Nrs in Set	Yarn	Encapsulation Material	Additional Transition Material(s)
1a, 1b, 1c, 1d	Yarn 1	Polyurethane-based potting compound	-
2a, 2b, 2c, 2d	Yarn 2	Polyurethane-based potting compound	-
3a, 3b, 3c, 3d	Yarn 1	Polyurethane-based potting compound	Laminated knit fabric wrapped around narrow fabric
4a, 4b, 4c, 4d	Yarn 2	Polyurethane-based potting compound	Laminated knit fabric wrapped around narrow fabric

## Data Availability

Not applicable.

## References

[1] Farringdon J., Moore A.J., Tilbury N., Church J., Biemond P.D. Wearable sensor badge and sensor jacket for context awareness. Proceedings of the Digest of Papers. Third International Symposium on Wearable Computers.

[2] Mann S. (1997). “Smart clothing”: Wearable multimedia computing and “personal imaging” to restore the technological balance between people and their environments. Proceedings of the Fourth ACM International Conference on Multimedia.

[3] Mann S. (1996). Smart clothing: The shift to wearable computing. Commun. ACM.

[4] Mann S. (1997). Smart clothing: The wearable computer and wearcam. Pers. Technol..

[5] De Pasquale G., Mura A. (2018). Accelerated lifetime tests on e-textiles: Design and fabrication of multifunctional test bench. J. Ind. Text..

[6] Zaman S.U., Tao X., Cochrane C., Koncar V. (2021). Wash Analyses of Flexible and Wearable Printed Circuits for E-Textiles and Their Prediction of Damages. Electronics.

[7] de Vries H., Cherenack K.H. (2014). Endurance behavior of conductive yarns. Microelectron. Reliab..

[8] Zysset C., Cherenack K., Kinkeldei T., Tröster G. (2010). Weaving integrated circuits into textiles. International Symposium on Wearable Computers (ISWC) 2010.

[9] Cherenack K., Pieterson L.V. (2012). Smart textiles: Challenges and opportunities. J. Appl. Phys..

[10] Bolton W., Bolton W. (2015). Chapter 4-I/O Processing. Programmable Logic Controllers.

[11] Gonzalez M., Axisa F., Bulcke M.V., Brosteaux D., Vandevelde B., Vanfleteren J. (2008). Design of metal interconnects for stretchable electronic circuits. Microelectron. Reliab..

[12] Sivaramakrishnan K., Theodore N.D., Moulder J.F., Alford T.L. (2009). The role of copper in ZnO/Cu/ZnO thin films for flexible electronics. J. Appl. Phys..

[13] Schischke K., Nissen N.F., Schneider-Ramelow M. (2019). Flexible, stretchable, conformal electronics, and smart textiles: Environmental life cycle considerations for emerging applications. MRS Commun..

[14] Koshi T., Nomura K.-I., Yoshida M. (2021). Measurement and analysis on failure lifetime of serpentine interconnects for e-textiles under cyclic large deformation. Flex. Print. Electron..

